# Transcriptomic responses in mouse blood during the first week after *in vivo* gamma irradiation

**DOI:** 10.1038/s41598-019-54780-0

**Published:** 2019-12-04

**Authors:** Sunirmal Paul, Norman J. Kleiman, Sally A. Amundson

**Affiliations:** 1 Center for Radiological Research, Vagelos College of Physicians and Surgeons, Columbia University Irving Medical Center, New York, NY 10032 USA; 2 Department of Environmental Health Sciences, Mailman School of Public Health, Columbia University Irving Medical Center, New York, NY 10032 USA

**Keywords:** Cell signalling, Transcriptomics, Predictive markers

## Abstract

Due to limitations of available human models for development of gene expression based radiation biodosimetry, many such studies have made use of mouse models. To provide a broad view of the gene expression response to irradiation in the mouse, we have exposed male C57BL/6 mice to 0, 1.5, 3, 6 or 10 Gy of gamma rays, sacrificing groups of the mice at 1, 2, 3, 5, or 7 days after exposure. We then profiled global gene expression in blood from individual mice using Agilent microarrays. In general, we found increasing numbers of genes differentially expressed with increasing dose, with more prolonged responses after the higher doses. Gene ontology analysis showed a similar pattern, with more biological processes enriched among the genes responding to higher doses, and at later times after exposure. Clustering the timecourse expression data using maSigPro identified four broad patterns of response, representing different gene ontology functions. The largest of these clusters included genes with initially decreased expression followed by increased expression at later times, a pattern of expression previously reported for several genes following neutron exposure. Another gene cluster showing consistent down regulation suggests genes useful for biodosimetry throughout the first week after exposure can be identified.

## Introduction

There continues to be worldwide concern about potential human health risks to large populations from radiation exposure after a nuclear accident or terrorist incident; e.g., detonation of a “dirty bomb” or an improvised nuclear device. Current research is directed at developing both radiation countermeasures and methods using biomarkers to better quantify which individuals might best benefit from therapeutic interventions^1–4^. Biodosimetry approaches range from improving and automating classical cytogenetic methods^5,6^ to developing novel methods based on high-content screening, such as transcriptomics, proteomics, and metabolomics^7–10^.

Our group, and others, have focused on the development of transcriptomic signatures derived from whole blood, a highly radiation responsive tissue requiring minimally invasive collection methods. Some studies have used human material, mainly blood from patients undergoing total body irradiation (TBI) prior to bone marrow transplantation^11–14^, or blood from healthy donors irradiated *ex vivo*^15–20^. The TBI model is limited by clinical irradiation protocols, which provide a narrow range of doses, usually delivered in several fractions, and by concerns that at a molecular level, the radiation response of extremely ill patients does not accurately reflect that of healthy individuals. While *ex vivo* irradiation of blood drawn from healthy donors provides flexibility in the doses studied, this model only partially recapitulates the *in vivo* response^11,13^, and the blood has a limited useful lifetime in culture^21^. The elapsed time after exposure is an important consideration for the development of transcriptomics-based radiation signatures, as gene expression changes in response to irradiation are highly dynamic^22^. Much biodosimetry signature development has therefore used mouse models to provide *in vivo* responses at a range of doses and exposure times^12,14,23–27^, however, most such studies reporting whole-genome expression data have focused on limited post-irradiation time points.

Here we report the first broad analysis of whole-genome gene expression in the blood of mice during the first week after exposure to gamma radiation doses from 0–10 Gy. The study covers the initiation of biological responses and the time period of most interest for radiation biodosimetry or triage. While the data themselves will be useful for signature development, this paper focuses on the temporal patterns of gene expression response and the nature of the biological and molecular functions implied by the patterns of gene expression change. The study provides a foundation for further studies of the role of gene expression changes during the first week of response after exposure to a range of sub-lethal and lethal doses of ionizing radiation.

## Results

### Differential gene expression in mouse blood after irradiation

**Figure 1:**
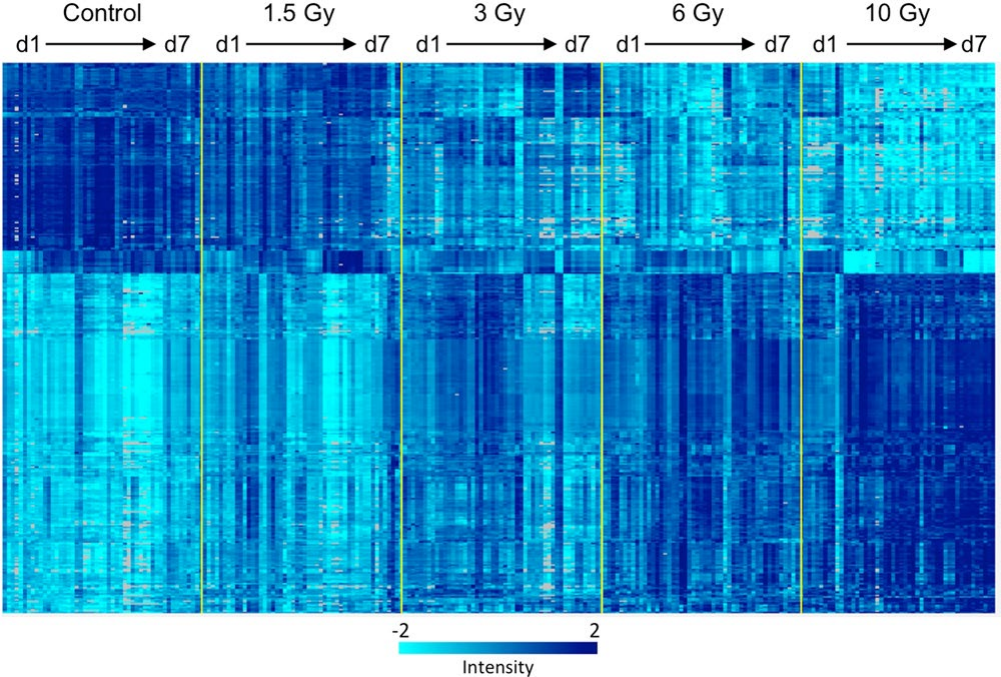
Heatmap of the expression of the 381 genes (rows) that were significantly differentially expressed throughout the first week after exposure to any of the doses of radiation tested. Intensity of expression in individual mice (columns) was measured by microarray and is indicated according to the color scale, with light cyan representing the lowest level of expression, and dark blue the highest level of expression. Grey indicates an expression level not significantly above the microarray background. The annotated list of genes in clustered order is available in Supplementary File 2.

The numbers of genes differentially expressed at each sacrifice time (1, 2, 3, 5, or 7 days) and γ-ray dose (1.5, 3, 6, and 10 Gy), along with their false discovery rate (FDR), are summarized in Table 1. The full gene lists with statistics and fold-change data are available in Supplementary File 1. Of these genes, we found that 381 were differentially expressed at all times measured within a week after exposure to at least one of the doses tested (Fig. 1).

**Table 1:** Differentially expressed genes by dose and time.

Days post irradiation	1.5 Gy	3 Gy	6 Gy	10 Gy
1	97 (75%)*	1337 (65%)	1462 (56%)	1308 (45%)
2	271 (18%)	4482 (42%)	5671 (35%)	5526 (40%)
3	86 (42%)*	4950 (39%)	7506 (44%)	8181 (45%)
5	488 (92%)	1648 (50%)	5706 (43)%	8763 (42%)
7	14 (29%)*	540 (34%)	3623 (39%)	8871 (40%)

The number of genes significantly differentially expressed relative to time matched controls at the level of p < 0.001 at each dose and time is listed along with the percentage of up-regulated genes, which is given in parentheses.

^*^All genes had a false discovery rate below 5%, with the exception of the groups marked with an asterisk. See Supplementary File 1 for full gene lists.

Since gene expression measurements made from whole blood reflect the response of multiple cell types with different radiation sensitivities, some researchers have isolated specific lymphocyte subtypes for the development of radiation biodosimetry signatures^28,29^. We have chosen to work with whole blood in part to reduce the pre-processing steps that would be needed to bring an assay to the point of care. This does mean that the gene expression changes we find may be partially driven by changes in the relative mixture of cell types in circulation after different times and exposure doses. We used Cibersort^30^ to deconvolute our gene expression data and estimate the relative abundance of different cell subtypes. The cell-type signatures used in Cibersort are based only on gene expression data from non-irradiated cells, however, and cannot distinguish between changes in cell numbers and functional changes during the response to radiation. Nonetheless, Cibersort indicated a dose-dependent depression in the representation of naïve B cells throughout the experiment, compensated to some extent by an increase in the inferred number of macrophages after exposure (Supplementary File 1). As biomarkers of radiation effects are developed it may be of interest to further pursue the cellular source of the signals through cell sorting experiments.

### Gene ontology analysis

**Figure 2:**
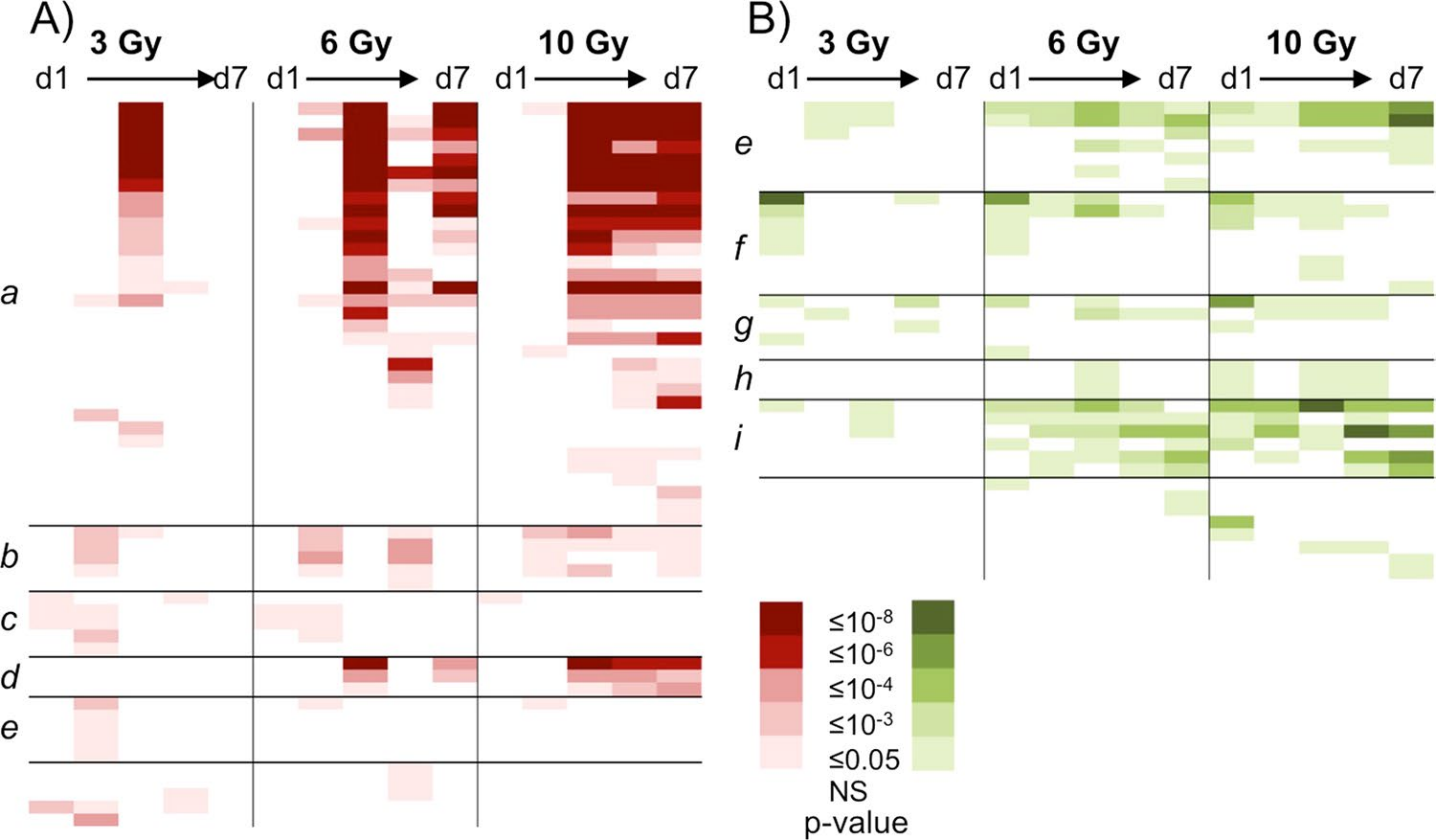
Gene Ontology analysis of differentially expressed genes. PANTHER was used to identify significantly over-represented GO-Slim biological processes (rows) among (**A**) up- or (**B**) down-regulated genes at 1, 2, 3, 5, or 7 days after exposure to 3, 6, or 10 Gy gamma radiation. Each column represents a dose and time combination. To illustrate the patterns of response among biological processes, the cells are colour coded by p-value as shown in the key. Related processes have been grouped together in the marked regions: (a) metabolic processes, (b) transport/localization, (c) adhesion/movement, (d) mitochondrial processes, (e) signalling/communication, (f) immune response, (g) cell cycle, (h) cell death, (i) differentiation/development. Details including the process names and p-values are available in Supplementary File 2.

We used the PANTHER Statistical Overrepresentation Test^31^ to identify biological processes that were significantly enriched (p < 0.05 with the fairly stringent Bonferroni correction for multiple comparisons) within the sets of up- or down-regulated genes at the various times after irradiation. The patterns of significant enrichment of biological processes among genes responding to 3, 6 or 10 Gy γ-rays are summarized in Fig. 2 as a function of time since exposure, with the actual processes listed in Supplementary File 2. No significantly enriched biological processes were found among the genes responding to 1.5 Gy γ-rays. We found many of the same processes enriched among the genes responding to the three higher doses, but with a shift in the timing of the perturbation dependent on dose. This included many processes related to metabolism among the up-regulated genes, and functions such as signal transduction, immune system process, and cell cycle among down-regulated genes. In general, the perturbation of processes was more quickly resolved after lower doses and more prolonged after the higher doses. Functions related to cell death only appeared as significantly enriched after doses of 6 Gy and above, possibly due to the stringency of the multiple comparison correction applied in the analysis.

Further analysis using a less stringent FDR correction for the multiple comparisons applied using ToppFun^32^ revealed additional enriched biological processes among the lower doses (Supplementary File 2). In the ToppFun analysis, many cell death processes were identified as significantly enriched following both 1.5- and 3-Gy doses. ToppFun also revealed additional enriched processes among the genes responding to the 1.5-Gy exposures, particularly during the first 3 days after exposure. These included immune and inflammatory processes, signalling, and apoptosis and cell death processes, consistent with the results of the more stringent analysis of the higher-dose gene sets.

### Prediction of upstream regulators of radiation response genes

**Figure 3:**
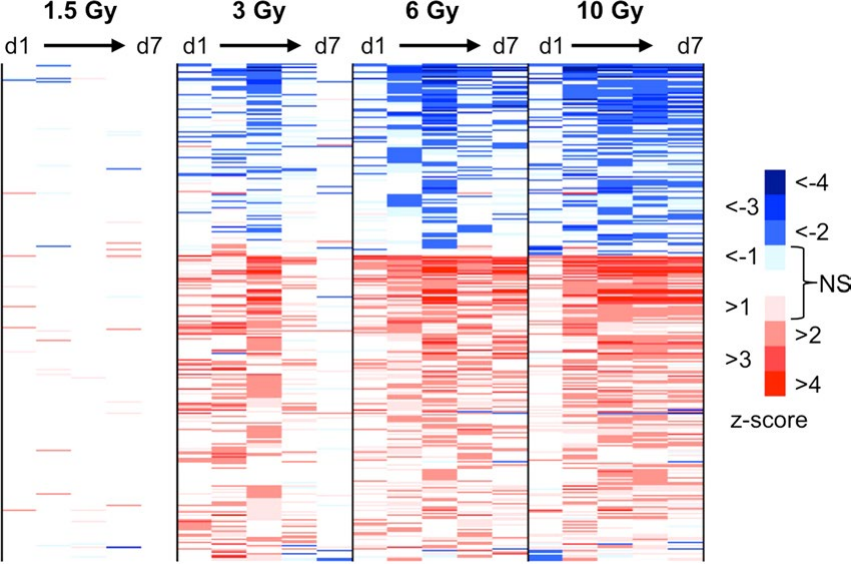
Predicted upstream regulator activation and inhibition. Significant gene expression fold-changes relative to time-matched controls were analysed separately for each dose and time combination (columns) using Ingenuity Pathway Analysis (IPA) to predict changes in upstream regulatory factors (rows). Results are coloured by z-score according to the key. Z-scores between 1 and 2 or −1 and −2 are also coloured to help visualize trends, but these are not deemed significant (NS). The annotated list of regulators with significant prediction of activation (red) or inhibition (blue) and their associated z-scores is available in Supplementary File 2.

We also used the core analysis function in Ingenuity Pathway Analysis^33^ to predict changes in the activity of upstream regulators that might contribute to the gene expression response to radiation. A significant z score (z > 2 or z < −2) was obtained for 307 regulators (Fig. 3, Supplementary File 2), with most showing a consistent direction of predicted activity change across time. As with the downstream biological processes, we found broadly similar patterns among the upstream regulators predicted to respond to the different radiation doses, with generally more long-lived responses after the higher doses. In general, regulators were significantly activated or suppressed at only one or two times after the lowest dose, showing more persistence after higher doses.

### Clustering of gene expression profiles across time

**Figure 4:**
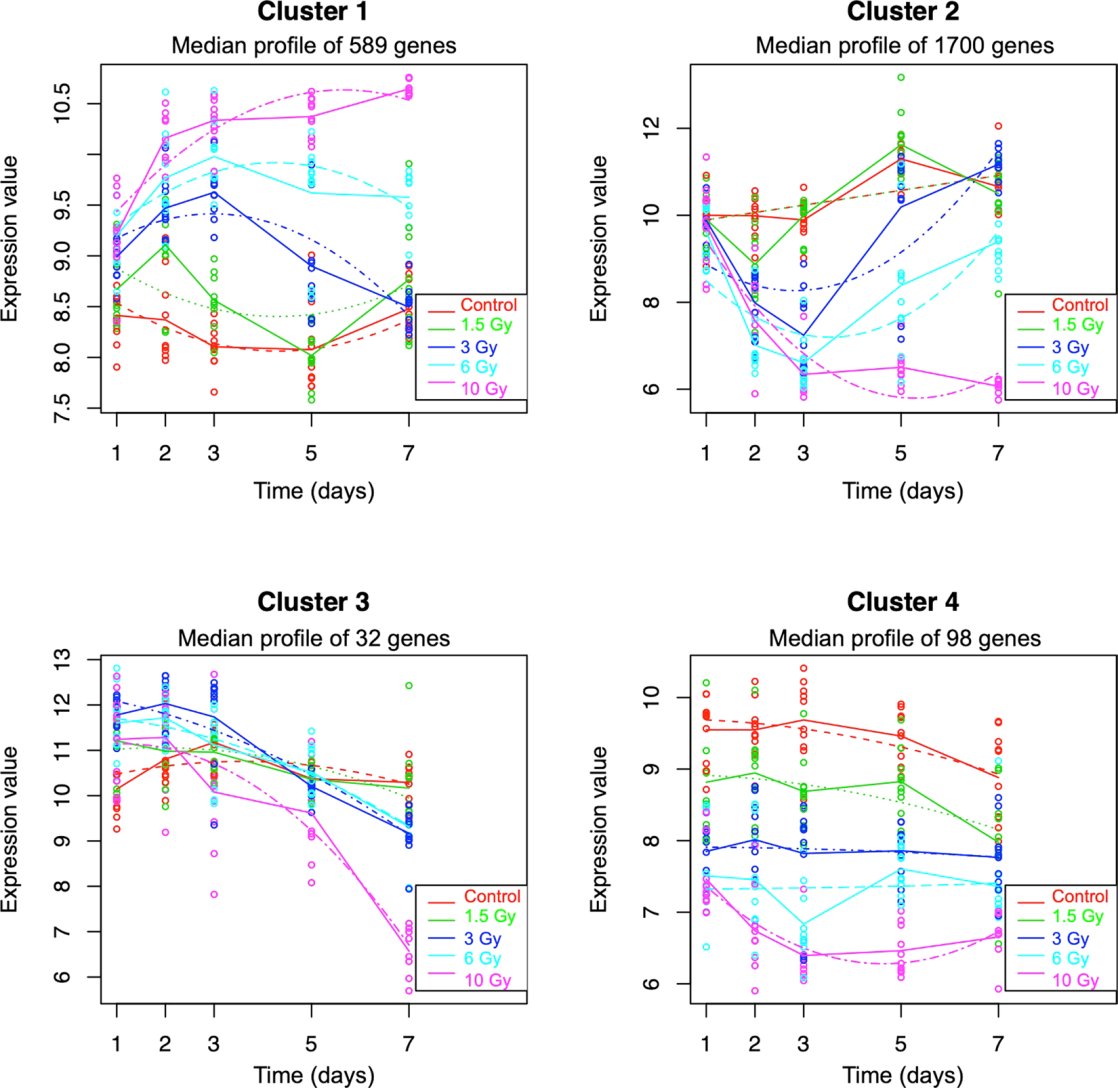
Gene expression profile clusters. The maSigPro R package was used to identify genes with significant changes in expression over time, and with significant differences in response as a function of dose. The gene expression profiles were grouped into four clusters that showed distinct temporal profiles, and the median expression of all genes in the cluster was plotted for each dose and time. Solid lines connect the average expression to show the trends for each dose group, and the dashed lines show the regression curves fitted to the data. Gene lists and ontology analysis for each cluster are available in Supplementary File 3.

We also used maSigPro^34^ to independently identify genes with significantly different expression profiles as a function of time after radiation exposure, based on quadratic regression models. The analysis identified 2419 genes with expression profiles significantly different from controls (FDR < 0.05) after any of the doses tested (Supplementary File 3). These were sorted into four clusters with different response profiles across the time course (Fig. 4). Increasing the number of clusters above four did not reveal any new distinct patterns of expression. We then applied the PANTHER Statistical Overrepresentation Test^31^ to the genes in each cluster to identify biological processes or pathways enriched within each of the profiles (Supplementary File 3).

Cluster 1 consisted of 589 genes rapidly up-regulated after radiation exposure, with an increasing magnitude and duration of response as the dose increased. Genes with roles in the p53 pathway (Bonferroni-adjusted P = 0.024) and cell cycle-related functions, such as “SCF(Skp2)-mediated degradation of p27/p21” (Bonferroni-adjusted P = 0.019) and “Cyclin E associated events during G1/S transition” (Bonferroni-adjusted P = 0.046), as well as many signalling and metabolic functions, were over-represented in this cluster.

**Figure 5:**
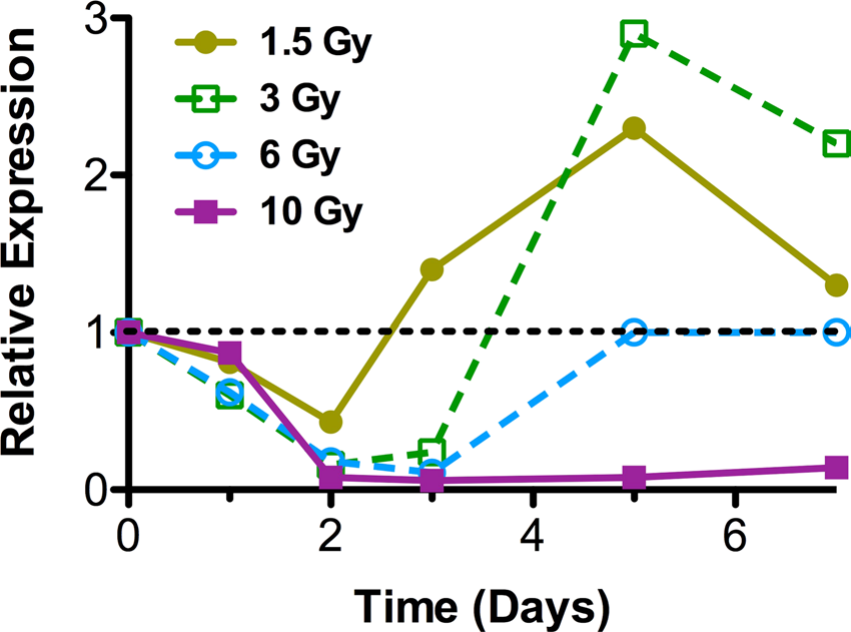
Expression of Ccna2 across the timecourse as measured by microarray. Each point represents the mean ratio of Ccna2 expression normalized to time matched un-irradiated controls (n = 10; 10 Gy 7 days n = 8). The dotted line indicates control expression levels.

The largest cluster was Cluster 2, with 1700 genes down-regulated at earlier time points. Similar to Cluster 1, these genes also showed a larger magnitude and duration of response with increasing dose. Pathways related to protein translation and ribosomes were among those over represented in Cluster 2. We looked more closely at the expression ratios of one of the Cluster 2 genes, *Ccna2*, as a function of dose and time (Fig. 5). This gene illustrates the pattern of early down regulation followed by a recovery that takes longer the higher the dose, as reflected in the summary plot of the Cluster 2 pattern (Fig. 4). However, it also reveals that at lower doses, the fairly early recovery from reduced expression is followed by an over-shooting of the control levels, with a temporary increase in expression, again resolving towards baseline more rapidly at the lowest dose.

The smallest cluster, Cluster 3, had only 32 genes. These showed a trend of slight up-regulation at early times after irradiation, without a clear dose effect, and a trend of down-regulation at later times, particularly after the highest dose. Despite the small number of genes in this cluster, hedgehog signalling and several other signalling pathways were over represented. Cluster 4 consisted of 98 genes with a fairly constant down regulation at all times after exposure, and a trend of greater magnitude of effect with increasing dose. Processes significantly over represented among the genes of Cluster 4 included calcium ion homeostasis, apoptosis, and immune processes.

## Discussion

We used male C57BL/6 mice as a model to delineate the gene expression changes occurring during the first week after exposure to a broad range of acute ionizing radiation doses. In general, higher doses resulted in greater numbers of responding genes, larger magnitudes of response, and a more sustained gene expression response (Table 1, Supplementary File 1). It should be noted that at later times, gene expression reflects the outcome of multiple signal transduction cascades, and should be considered more a reflection of the current physiological state of the animals than a direct response to radiation exposure.

It should also be noted that while the mouse model gives us the flexibility to perform a study of dose and time dependence of gene expression *in vivo*, results from mouse cannot be directly applied to humans. Not all of the same genes are differentially expressed in both species following radiation exposure. Moreover, some genes show opposite directions of differential expression in mice and humans^35^, further complicating translation between species. Comparison of the genes found differentially expressed after 24 h in the present mouse study with those identified in Ghandhi *et al*.^35^ as responding in the opposite direction in human and mouse yielded from 2–16 genes in common depending on the dose (Supplementary File 1). Comparison with the genes responding in the same direction in human and mouse showed from 7–80 genes in common (Supplementary File 1), suggesting a conserved core of response. Even data from non-human primate experiments provides much better discrimination of dose in human samples when careful gene selection and a translation algorithm are applied^36^. Nonetheless, studies in mice can provide an overview of the evolution of gene expression responses over time after irradiation, and help to inform the design of experiments aimed at signature translation.

Among the genes differentially expressed during the response to radiation, we identified 381 that were differentially expressed relative to controls at all times throughout the course of the study following at least one exposure dose. Visualizing these most consistently responsive genes as a heat map (Fig. 1) revealed patterns of expression dependent on both dose and time, with the response to lower doses showing smaller magnitudes of change and more rapid resolution towards control levels compared to the responses to higher doses. The very dynamic nature of gene expression highlighted here, and the difference in duration of many responses as a function of dose, are issues that must be addressed as part of the gene selection and algorithm development for practical radiation biodosimetry.

Gene ontology analysis of the set of 381 sustained response genes using PANTHER indicated significant enrichment of pathways and processes related to immune functions, as well as apoptotic processes and p53 signalling (Supplementary File 2). These processes have previously been shown to be prominent in both the mouse and human response to radiation during the first week after *in vivo* exposure, with similar patterns of increasing duration of effect at increasing doses^13,25,37–39^.

Taking a more granular look at the biological processes enriched among the significantly differentially expressed genes at each time and dose point revealed a diversity of responses dependent on dose and time since exposure. Among up-regulated genes, processes related to metabolism and catabolism were over-represented after doses of 3 Gy and above, consistent with results of earlier x-ray studies in C57BL/6 mice 1 day after a 4-Gy dose or 7 days after a 3-Gy dose^37,38^. Additional processes related to mitochondrial function and protein metabolism became significantly enriched among up-regulated genes following exposure to doses of 6 and 10 Gy, with even more metabolic processes emerging as significantly enriched among genes responding to 10 Gy on day 3 and later. To put these responses into context, the LD_50/30_ for the mice used in this study (the dose at which half the animals exposed will die within a month) is around 8 Gy, so that the 6-Gy dose represents significant but survivable radiation damage, while the 10-Gy dose would be uniformly fatal within about 2 weeks of exposure.

Among down-regulated genes, processes related to immune functions, proliferation and differentiation, and signalling were enriched after doses of ≥3 Gy, with additional developmental, signalling, cell death, immune and metabolic processes becoming significantly over-represented after higher doses. Immunity related functions are also prominent among down-regulated genes in a number of previously published radiation studies, including 24 h after the start of total body irradiation in cancer patients^13^ and 1 and 7 days after a 3- or 4-Gy x-ray dose in mice^37,38^. The effect on the immune system and reduced expression of immune-related genes appears to be relatively long lived, as significant enrichment of these processes was reported to last at least from day 2 through day 30 after whole-thorax lung irradiation of non-human primates^40^, and through the same time period after gamma-ray exposure from internally deposited ^137^Cs in C57BL/6 mice^25^.

We also used the upstream analysis feature of IPA to predict the activity of upstream regulators after radiation exposure based on the observed changes in gene expression. As summarised in Fig. 3 and Supplementary File 2, many upstream factors were predicted as potentially involved in the radiation response. Familiar radiation responses were found among the transcription factors that were implicated as regulators after all doses, such as activation of Trp53 and suppression of Myc. The vast majority of upstream factors were predicted as either activated or suppressed, although a few, such as IFNG, showed significant predictions of both activation and suppression at different times after multiple doses of radiation. A similar reversal of IFNG activity direction was also predicted in an earlier study, in which non-human primates were exposed to 10 Gy x-ray to the whole thorax^40^. This apparent fluctuating of activity over time may represent a feedback mechanism that plays a role in regulation and maintenance of the prolonged impact on genes related to immune functions seen following radiation exposure, and would be an interesting area for further study.

Reversal of the direction of expression relative to controls has also been reported for individual genes following irradiation. For instance, in a study comparing gene expression responses of mice to x-ray and neutron exposure^38^, 14 genes (*Mta3, Eif5, Mtmr3, Wdr26, Anp32e, Ubac1, Bsdc1, E2f2, Slc25a51, Ube2c, Fzr1, Ccna2, Cdc25b*, and *Nusap1*) were reported to be down regulated at one day after exposure and up regulated at 7 days after exposure. Of those genes, all but one (*Slc25a51*) were identified by our maSigPro analysis as differentially expressed over time, and were members of Cluster 2, which showed initial suppression followed by recovery of transcript levels by 7 days after irradiation in mice exposed to doses less than 10 Gy (Fig. 4).

We looked more closely at the expression pattern of *Ccna2* (Fig. 5), one of the genes from the earlier study, and found the response to gamma-rays was consistent with the reported behaviour of this gene at days 1 and 7 after a 1- or 4-Gy dose of x-rays or a 1-Gy dose of neutrons^37^. It is possible that had we continued our experiment for a longer period of time, the 6-and 10-Gy doses may also have shown a similar pattern of over expression of this gene at later times, or this may be a response that only occurs in a fairly narrow dose range. Visualizing the genes in Cluster 2 as a heatmap (Supplementary File 3) reveals a similar pattern across the cluster. From the heatmap it appears that in general these genes show the clearest switch between under and over expression following the 3-Gy dose, and suggest that directional switching or overshooting of expression levels may be more widespread in the response to radiation than suggested by the small number of genes previous reported to show this pattern of expression^38^, with a strong dependence on both dose and time since exposure.

In contrast to the very complex gene expression patterns evident in Cluster 2, the genes in Cluster 4 show a relatively simple and stable pattern of dose-dependent decreased expression across the seven days of the experiment. This pattern is quite similar to that of reduced B-cell numbers inferred from the Cibersort analysis (Supplementary File 1). A sustained loss of B cells in circulation or suppression of their specialized functions would also be consistent with the gene ontology results of the Cluster-4 genes (Supplementary File 3), suggesting that a loss of B cells may be responsible for the apparent loss of expression of these genes. While flow cytometric analysis will be needed to confirm the source of the Cluster-4 gene pattern, these genes are attractive for biodosimetry applications. They could potentially give the same dose readout at multiple days after exposure, avoiding the need for separate gene sets or a time-since-exposure correction factor built into the dose algorithm. Detailed informatic analysis coupled with extensive independent experimental validation will still be required to account for different time- and dose-dependent gene expression patterns and establish robust gene expression biodosimetry approaches.

## Methods

### Animals and irradiations

Animal experiments were conducted in accordance with applicable federal and state guidelines and were approved by the Columbia University Institutional Animal Care and Use Committee (IACUC) under protocol number AC-AAAB8465. Approximately two-month-old male C57BL/6N mice (stock number 027) were obtained from Charles River Labs (Frederick, MD) and acclimatized for at least a week before whole body irradiation. At 8–10 weeks of age, mice were exposed in the early afternoon to radiation doses of 0, 1.5, 3, 6, or 10 Gy using an AECL Gammacell-40 ^137^Cs γ-ray source (Ottowa, CA) at a dose rate 1.05 Gy/ min. During exposure, animals were individually housed within a RadDisk™ Rodent Microisolation Irradiator Disk (Braintree Scientific, Braintree, MA), which facilitates simultaneous uniform exposure of up to eight mice.

### RNA isolation and microarray hybridization

For each dose, 10 mice were humanely euthanized by CO_2_ asphyxiation at 1, 2, 3, 5 and 7 days after irradiation. Approximately 0.8–1.0 ml blood was obtained immediately from each animal via cardiac puncture and added to 1.5 ml PAXgene Blood RNA stabilization and lysis solution (Qiagen, Germantown, MD) in a 15 ml conical centrifuge tube and gently inverted several times. Additional blood RNA stabilization/lysis solution was added to achieve a 1:5 final ratio of blood:PAXgene solution and the samples were kept at room temperature for two hours prior to overnight refrigeration. The next day, RNA was purified according to the manufacturer’s instructions using a PAXgene Blood RNA kit (Cat. No. 762164).

Globin coding transcripts were subsequently reduced using a GLOBINclear™ kit (Ambion Technologies AM1981), RNA quantified using a Nanodrop 2000 (ThermoFisher Scientific) and RNA quality monitored using a Bioanalyzer 2100 (Agilent Technologies, Santa Clara, CA). RNA samples were routinely obtained with RNA integrity numbers (RIN) of 8.5 or greater.

Individual RNA samples were obtained from 10 different mice for all time points and harvest times with the exception of 7 days after exposure to 10 Gy. Notably, at this highest dose and latest post-exposure sampling, RNA yields from each animal were highly variable and considerably lower than expected. Six animals from this exposure group also required euthanasia prior to 7 days post-exposure due to radiation-induced morbidity, and were removed from the study. In total, more than 20 animals were exposed to 10 Gy for the 7-day time point, yielding only 8 samples with sufficient RNA quality and yield for microarray hybridization.

Cyanine-3 labeled cRNA was produced, fragmented, and hybridized to whole genome Agilent Mouse Gene Expression 4 × 44 K v2 Microarrays (G4846A) as previously described^41^. Slides were scanned using default parameters on an Agilent DNA microarray scanner (G2505B) and data were extracted from the image files using Agilent Feature Extraction software with default parameters for background correction and non-uniform feature flagging.

### Data analysis

Hybridization intensities were imported into BRB-ArrayTools (v 4.5.1)^42^, where they were log_2_-transformed and median normalized. Features not significantly above background intensity or flagged as non-uniform outliers on 25% or more of the microarrays were excluded, with 19220 features passing the filter. Invariant features, defined as those not showing ≥1.5-fold change in a minimum of 20% of the hybridizations, were also excluded from analysis, resulting in 17920 features that were used for class comparison. The microarray data are available through the Gene Expression Omnibus^43^ using Accession Number GSE124612 (https://www.ncbi.nlm.nih.gov/geo/query/acc.cgi?acc=GSE124612). For each dose, the Class Comparison feature of BRB-ArrayTools was used to identify genes that were differentially expressed (p < 0.001) relative to time matched controls at each of the sampled times after exposure. The method of Benjamini and Hochberg^44^ was used to estimate FDR to control for false positives (FDR < 5%). The Dynamic Heatmap Viewer feature of BRB-ArrayTools was used to cluster genes using a one minus correlation and average linkage.

Gene ontology enrichments of gene sets were determined using the default parameters of the Statistical Overrepresentation Test in PANTHER (Protein ANalysis THrough Evolutionary Relationships) Tools^45^. GO-Slim Biological Processes with Bonferroni corrected p values < 0.05 were reported as significantly enriched. PANTHER and Reactome pathways (Bonferroni corrected p values < 0.05) were also reported for the analysis of the maSigPro gene clusters. ToppFun^32^ analysis using the default settings was also applied to the 1.5- and 3-Gy results, as this software allows use of the less stringent FDR approach to correct for multiple comparisons. Biological processes with FDR < 5% were reported as significantly enriched.

The lists of significantly differentially expressed genes from each dose and time point along with their intensity measurements were also uploaded for analysis using Ingenuity Pathway Analysis (QIAGEN Inc., https://www.qiagenbioinformatics.com/products/ingenuity- pathway-analysis)^33^. Putative upstream regulators of the observed gene expression responses were predicted in the IPA core analysis, with a comparison analysis being run to compare results across the dose- and time-course. IPA predicts activation or inhibition of regulatory factors upstream of a set of gene expression changes based on a database of relationships between gene products drawn from the published literature. A z-score ≤−2 or ≥2 was required to report a prediction of inhibition or activation (respectively) as statistically significant.

MaSigPro^34^ was applied to the filtered dataset using default parameters and a quadratic regression model to first identify genes with significant expression differences from controls (FDR <0.05), and then to identify significant differences in their time-dependent profiles across the 7-day timecourse using backward step-wise regression with a p < 0.05 statistical cut off. The significant genes were then clustered into 4 patterns for visualization and further analysis.

Cibersort^30^ was applied to the un-filtered dataset using default parameters and the cell-type signature matrix derived from C57BL/6 mice by Chen *et al*.^46^ to infer changes in the relative numbers of cell sub-types after different times and exposure doses.

## Supplementary information

Supplementary File 1

Supplementary File 2

Supplementary File 3
